# Effect of concentration and duration of particulate matter exposure on the transcriptome and DNA methylome of bronchial epithelial cells

**DOI:** 10.1093/eep/dvaa022

**Published:** 2021-02-28

**Authors:** Steven K Huang, Priya Tripathi, Lada A Koneva, Raymond G Cavalcante, Nathan Craig, Anne M Scruggs, Maureen A Sartor, Furong Deng, Yahong Chen

**Affiliations:** 1 Division of Pulmonary and Critical Care Medicine, Department of Internal Medicine, University of Michigan Medical School, 6301 MSRB III, 1150 W Medical Center Drive, Ann Arbor, MI 48109, USA; 2 Department of Computational Medicine and Bioinformatics, University of Michigan Medical School, Ann Arbor, Room 2017, Palmer Commons 100 Washtenaw Avenue Ann Arbor, MI 48109-2218, USA; 3 Epigenomics Core, University of Michigan, Ann Arbor, Medical Science Research Building II Rm C568 1150 W. Medical Center Dr Ann Arbor, MI 48109, USA; 4 Department of Occupational and Environmental Health Sciences, School of Public Health, Peking University, Xueyuan Road 38, Haidian District, Beijing, China; 5 Department of Respiratory Medicine, Peking University Third Hospital, No. 49, Huayuan North Road, Haidian District, Beijing, China

**Keywords:** BEAS-2B, DNA methylation, RNA-Seq, PM2.5, TET, RRBS

## Abstract

Exposure to particulate matter (PM) from ambient air pollution is a well-known risk factor for many lung diseases, but the mechanism(s) for this is not completely understood. Bronchial epithelial cells, which line the airway of the respiratory tract, undergo genome-wide level changes in gene expression and DNA methylation particularly when exposed to fine (<2.5 µm) PM (PM_2.5_). Although some of these changes have been reported in other studies, a comparison of how different concentrations and duration of exposure affect both the gene transcriptome and DNA methylome has not been done. Here, we exposed BEAS-2B, a bronchial epithelial cell line, to different concentrations of PM_2.5_, and compared how single or repeated doses of PM_2.5_ affect both the transcriptome and methylome of cells. Widespread changes in gene expression occurred after cells were exposed to a single treatment of high-concentration (30 µg/cm^2^) PM_2.5_ for 24 h. These genes were enriched in pathways regulating cytokine–cytokine interactions, Mitogen-Activated Protein Kinase (MAPK) signaling, PI3K-Akt signaling, IL6, and P53. DNA methylomic analysis showed that nearly half of the differentially expressed genes were found to also have DNA methylation changes, with just a slightly greater trend toward overall hypomethylation across the genome. Cells exposed to a lower concentration (1 µg/cm^2^) of PM_2.5_ demonstrated a comparable, but more attenuated change in gene expression compared to cells exposed to higher concentrations. There were also many genes affected by lower concentrations of PM_2.5_, but not higher concentrations. Additionally, repeated exposure to PM_2.5_ (1 µg/cm^2^) for seven days resulted in transcriptomic and DNA methylomic changes that were distinct from cells treated with PM_2.5_ for only one day. Compared to single exposure, repeated exposure to PM_2.5_ caused a more notable degree of hypomethylation across the genome, though certain genes and regions demonstrated increased DNA methylation. The overall increase in hypomethylation, especially with repeated exposure to PM_2.5_, was associated with an increase in expression of ten–eleven translocation enzymes. These data demonstrate how variations in concentration and duration of PM_2.5_ exposure induce distinct differences in the transcriptomic and DNA methylomic profile of bronchial epithelial cells, which may have important implications in the development of both acute and chronic lung disease.

## Introduction

Particulate matter (PM) remains one of the most harmful forms of air pollution, contributing to more than 3 million deaths per year worldwide (1). Traffic, diesel, and manufacturing are major sources of PM in industrialized cities and countries. Small particles, including those less than 2.5 µm in diameter (PM_2.5_), are especially harmful because of their ability to travel deep in the respiratory tract and cause toxicity to cells lining the airway. Over the decades, public health efforts have curtailed levels of PM_2.5_ in many countries throughout the world. However, recent studies have shown that low levels of exposure, even below that set by governmental regulatory agencies in many advanced countries, continue to cause harm in the general population (2, 3).

Bronchial epithelial cells line the airways and are the first cells that are exposed to PM_2.5_. Many studies have shown that PM_2.5_ causes oxidative stress and toxicity in bronchial epithelial cells in a concentration-dependent fashion (4–8). Several studies have also shown that PM_2.5_ causes widespread changes in the transcriptome of these cells (9–16). These studies, however, mostly employ PM_2.5_ at high concentrations and examine the effects of PM_2.5_ only after a single exposure and short time interval. How the transcriptome changes with lower concentrations and chronic exposure to PM_2.5_ has not been extensively studied. Recognizing that many humans are exposed to low levels of chronic air pollution, many investigators have attempted to model this phenomenon *in vitro* by treating epithelial cells with low levels of PM_2.5_ repeatedly over several days. These studies have shown that epithelial cells under these conditions persistently release inflammatory cytokines, develop epigenetic changes, and undergo epithelial–mesenchymal transition (17–19). Our laboratory has shown that repeated exposure to low-concentrations of PM_2.5_ over seven days resulted in the upregulation of many genes that were not observed after a single exposure to PM_2.5_ (20). However, a full transcriptomic analysis of this treatment protocol—along with a comparison with higher concentrations and short-term exposure—has not been reported. We hypothesize that even though a single exposure to high concentrations of PM_2.5_ may induce oxidative stress and DNA damage, along with reciprocal anti-oxidant and anti-toxin response, exposure to lower concentrations, especially over time, may cause more subtle changes in gene expression that might differ from exposure to higher concentrations, but play equally important roles in disease development.

DNA methylation is a well-characterized epigenetic mechanism recognized for its ability to influence gene expression, often in a persistent, heritable way. DNA methylation is critical to normal development, and alterations in DNA methylation have been shown to contribute to cancer (21), autoimmune disease (22), and asthma (23). DNA methylation, especially within gene promoters and in CpG islands, is traditionally associated with suppression of gene expression (24). More recent studies, however, have shown that increased DNA methylation, especially within gene bodies and exons, are associated with increased transcription (25), emphasizing the importance of distinguishing where in the gene DNA methylation changes are occurring and how it might affect gene transcription. PM_2.5_ has been shown in numerous *in vitro* and *in vivo* studies to cause changes in DNA methylation, either hyper- or hypo-, as measured in whole blood or in isolated cell types (26–28), though the significance of methylation changes at individual CpG loci is not always clear. Here, we examine the transcriptomic and DNA methylomic changes that occur in bronchial epithelial cells in response to PM_2.5_ at different concentrations and duration of exposure. We observed that PM_2.5_ causes widespread changes in the expression and DNA methylation of genes that are important in epithelial cell biology. That we observed distinct patterns of DNA methylomic and transcriptomic changes with different treatment protocols further highlight the importance of considering variables such as concentration and duration of exposure when trying to model and understand the pleiotropic effects of PM_2.5_.

## Methods

### Collection of PM_2.5_ and Extraction from Filters

PM_2.5_ was isolated from ambient air pollution collected in Beijing, China from 19–21 January 2015 as previously described (20). Low volume manual samplers placed on the rooftop of Peking University School of Public Health collected PM_2.5_ over a 24-h period on 90 mm Emfab filters, which are made of borosilicate fibers reinforced with woven cloth and bonded with polytetrafluoroethylene (TX40HI20WW, part #7234, Pall Company, Beijing Office, Beijing, China). Each filter was folded, wrapped in aluminum foil, and stored in −20°C until extraction. Twenty-four hours before extraction, filters were placed in sterile amber jars and equilibrated at fixed humidity and room temperature in a sterile biosafety containment hood, and weighed before wetting and extraction. To extract PM_2.5_, 20 ml of double distilled water was added to each amber jar and filters were sonicated (VWR, model no. 97043-968, VWR International, Radnor, Pennsylvania, USA) on ice at 15 min intervals for a total of 3 h. After sonication, filters were air-dried for 3 days in amber jars located in the same biosafety containment hood at constant humidity and room temperature before being weighed on a microbalance. The difference in weight (averaged from 3–5 measurements) before and after extraction was used to calculate the concentration (mg/ml). The extracted PM_2.5_ was aliquoted and stored for future use at −80°C.

### Cells and Treatments

BEAS-2B, a bronchial epithelial cell line, was cultured on collagen-coated tissue-culture plates in serum-free Bronchial Epithelial Growth Medium (BEGM; CC-3170, Lonza, Walkersville, MD), which consists of basal medium supplemented with standardized growth factors provided by the manufacturer (BEGM BulletKit CC-3171 and CC-4175; Lonza). Cells were maintained in a 37°C incubator with 5% CO_2_. Collagen-coated tissue-culture plates were prepared by coating plates with pre-made bovine collagen solution (PureCol-Type I Bovine Collagen Solution, Advanced BioMatrix, San Diego, CA) diluted to a concentration of 3 mg/ml with 0.1 N HCl overnight at 4°C. The liquid was then aspirated and plates were UV-irradiated for 30 min before they were washed three times with sterile water.

For experiments, cells were plated at a density of 5 × 10^5^ in six-well plates in BEGM and allowed to adhere overnight. Medium was replaced the next day with either fresh BEGM alone or fresh BEGM containing a low (1 µg/cm^2^) or high (30 µg/cm^2^) concentration of PM_2.5_ for 24 h. For experiments involving chronic, repeated exposure, cells were initially plated at a density of 2.5 × 10^5^ in six-well plates. Medium was replaced the next day with fresh BEGM either alone or with 1 µg/cm^2^ of PM_2.5_. This was repeated each day, with all cells being washed with phosphate-buffered saline (PBS) between treatments, for a total of six days before cell lysates were collected on day 7 for RNA and DNA analysis.

### RNA-Seq

RNA was initially isolated from cells using Trizol (Catalog Number 15596018, Invitrogen, Carlsbad, CA, USA); extracted RNA underwent an additional step for cleanup using the RNeasy kit (Qiagen, Germantown, MD). RNA was assessed for quality on the Agilent TapeStation and samples with RNA integrity number (RIN) > 7 were used for subsequent library preparation. Poly-A enrichment was used to select mRNA for library preparation, and samples were sequenced as a single-end 50 bp fragment on the Illumina HiSeq 4000, which was performed by the University of Michigan Advanced Genomics Core. Each experiment, including the single exposure protocol and repeated exposure protocol, was performed three independent times; a total of 15 samples were multiplexed and sequenced over two lanes.

### Enhanced Reduced Representation Bisulfite Sequencing (eRRBS)

Assessment of genome-wide DNA methylation was performed by eRRBS, as previously described (29). DNA was isolated from cells using DNeasy kit (Qiagen) and submitted to the University of Michigan Epigenomics Core for library construction and bisulfite sequencing. Briefly, DNA was fragmented using the restriction enzyme Msp I, end repaired, A-tailed, and ligated (all using reagents from New England Biolabs, Ipswich, MA, catalog #s: R0106M, M0203L, M0210L, M0212L) to pair-end methylated adapters from Illumina. DNA was then resolved on agarose gel and fragments (150–250 bp and 250–450 bp) were excised and eluted. Samples then underwent bisulfite conversion using the EZDNA Methylation kit (Zymo Research, Inc., Irvine, CA) and DNA amplified using the Roche High Fidelity FastStart system. Final libraries were cleaned using Agencourt AMPure beads before sequencing on the Illumina HiSeq 4000. Each experiment, including the single exposure protocol and repeated exposure protocol, was performed three independent times; a total of 15 samples were multiplexed and sequenced over five lanes. Raw data for all RNA-Seq and eRRBS samples were uploaded into the National Center for Biotechnology Information Gene Expression Omnibus (GEO) database under accession number GSE155617.

### Quantitative RT-PCR

RNA was reverse-transcribed to cDNA using the High Capacity cDNA Reverse Transcription Kit (Applied Biosystems, Carlsbad, CA, USA) according to the manufacturer’s recommendations; quantitative real-time PCR was performed on cDNA using SYBR green PCR Master Mix (Applied Biosystems) on a StepOne Real-time PCR System (Applied Biosystems). The fold-change in expression of target genes (purchased as pre-designed TaqMan assays with primer-probes from Applied Biosystems) was calculated by the ΔΔCt method relative to β-actin as the endogenous control. Glyceraldehyde-3-phosphate dehydrogenase (GAPDH) was used as an alternative endogenous control to verify the findings.

### Data Analysis

For RNA-Seq analysis, FASTQ raw reads were aligned using STAR (Spliced Transcripts Alignment to a Reference) version 2.5.3 to the reference genome GRCh38. Reads were counted using featureCounts and normalized using edgeR via the Bioconductor package. Each experiment was performed three independent times and the data from all three experiments was averaged together; differential expression relative to vehicle control was performed using edgeR and analyzed by glmQLFit with correction for multiple testing. A linear fold-change > 1.5 (log 0.6) with adjusted *P*-value (adjusted for false discovery rate) < 0.05 was deemed significant. For eRRBS analysis, sequencing reads were assessed for quality by FastQC, and reads were trimmed using TrimGalore to trim low-quality bases (quality score lower than 20), adapter sequences, and end-repair bases from the 3′ end of reads. Bismark was used for methylation calling and alignment (30) to the reference genome using Bowtie2 (31). The methylSig R package was used to identify sites of differential methylation (32). An absolute difference of > 10% methylation with adjusted *P*-value (adjusted for false discovery rate) < 0.05 was deemed significant.

Pathway analysis was performed using Advaita iPathway (Advaita Corportation, Ann Arbor, MI) analysis. Statistically significant pathways were defined by adjusted *P*-value < 0.05.

## Results

### Transcriptomic Changes in BEAS-2B Cells after Single Exposure of PM_2.5_

Other studies have reported the results of compositional analysis of PM_2.5_ from Beijing, China taken on the same or similar dates (January through February, 2015) as the PM_2.5_ that we used in our study (33, 34). In those analyses, PM_2.5_ was composed of anions ${\text{SO}}_{4}^{2-}$ and ${\text{NO}}_{3}^{-}$, elemental metals such as Ag, As, Cd, Cu, Hg, Pb, Se, and Zn, and polyaromatic hydrocarbons including Benz(a)anthracene, Chrysene, and 1,8-Naphthalic anhydride (33, 34). The largest source of this PM_2.5_ was identified as coming from vehicle emissions. The level of endotoxin in our highest treatment condition (30 µg/cm^2^) was 0.433 EU/ml, as previously described (20).

Although treatment with high concentrations of PM_2.5_ (≥ 50 µg/cm^2^) induced apoptosis and cytotoxicity, as shown in our previous study, measures of lactate dehydrogenase release and cleaved poly-ADP ribosylation remained low at concentrations up to 30 µg/cm^2^ in our experimental system (20). Levels of *IL6*, *TNF*, and *TSLP* robustly increased at this concentration, so treatment with 30 µg/cm^2^ was chosen as the maximum concentration for our transcriptomic and methylomic studies.

**Figure 1:**
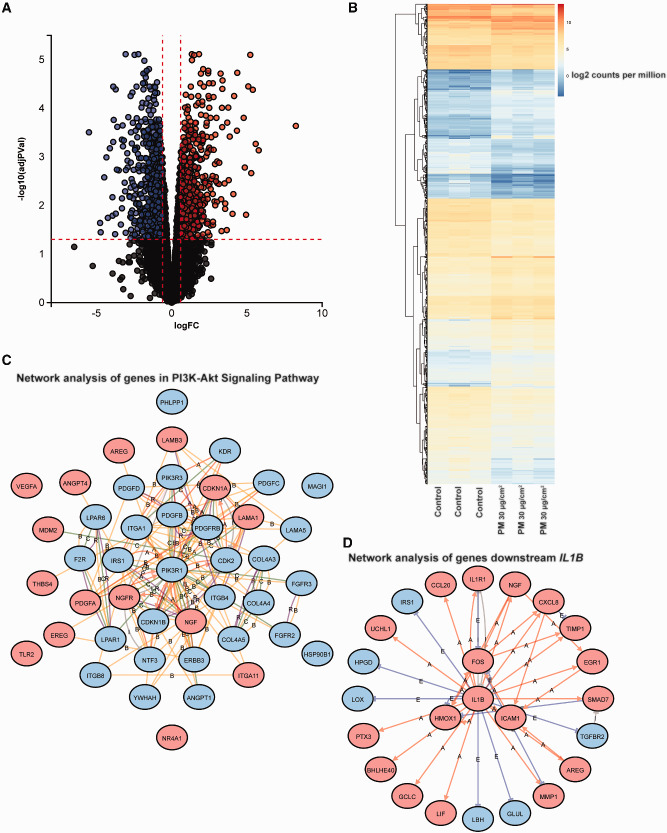
differential expression of genes after 24-h treatment with high-concentration PM_2.5_. (**A**) BEAS-2B cells were treated with 30 µg/cm^2^ of PM_2.5_ for 24 h before being analyzed by RNA-Seq. The volcano plot demonstrates expression of genes by log fold-change relative to adjusted *P*-value. (**B**) Heatmap of 1310 differentially expressed genes between control and treatment with 30 µg/cm^2^ of PM_2.5_. (**C**) Pathway analysis identified enrichment of genes within the PI3K-Akt Signaling Pathway. Shown is the network analysis of genes within this pathway, with red indicating upregulation and blue indicating downregulation after PM_2.5_ treatment. (**D**) Pathway analysis also identified *IL1B* as an upstream hub, with differentially expressed genes downstream of *IL1B* shown.

We first treated BEAS-2B cells with either vehicle control or 30 µg/cm^2^ of PM_2.5_ for 24 h and performed RNA-Seq analysis to examine the transcriptomic changes that occur with PM_2.5_ treatment. Compared to cells treated with vehicle control, cells treated with 30 µg/cm^2^ of PM_2.5_ demonstrated a statistically significant increase in 612 genes and decrease in 698 genes (Fig. 1). A list of all of the differentially expressed genes including fold-change and statistical significance is included in Supplementary Table S1. Examination of these differentially expressed genes included upregulation of *CYP1A1*, *CYP1B1*, and *AHRR*, which are commonly increased when cells have been exposed to polyaromatic hydrocarbons (7, 35), such as that present in urban PM_2.5_. Genes such as *HMOX1*, *NQO1*, *GCLM*, *SQSTM1*, and *FTL*, which are often increased in response to oxidative stress (36, 37), were also increased. Expression of *MDM2* and *CDKN1A* were also elevated after treatment with PM_2.5_, which was also expected as a result of the ability of high concentrations of PM_2.5_ to trigger DNA damage (5). The identification of these upregulated genes in our dataset, which have been shown individually in other studies, validates the reliability and accuracy of our global RNA-Seq analysis.

To take a more unbiased view of potential pathways enriched by these differentially expressed genes, we used Advaita to perform pathway analysis and identified pathways such as Cytokine–Cytokine Receptor Interaction, Glycine Serine Threonine Metabolism, mitogen-activated protein kinase (MAPK) Signaling Pathway, and PI3K-Akt Signaling Pathway that were enriched in our dataset (Table 1). Supplementary Table S2 lists the differentially expressed genes associated with these pathways. Although elevation of cytokines and enrichment of pathways such as MAPK and PI3K-Akt have been described in other studies of PM_2.5_ (9, 11, 12, 16), pathways related to amino acid synthesis and metabolism and cardiovascular disease (Table 1) were uniquely found in our dataset. Network analysis of genes within these pathways allowed us to identify key interactions, including genes that may serve as regulatory hubs (Fig. 1C, Supplementary Fig. S1).

**Table 1:** top pathways from differentially expressed genes in cells treated with PM_2.5_ (30 µg/cm^2^) vs. Control, 24 h

Pathway name	countDE^a^	countAll^a^	pORA_FDR^b^	pOverall_FDR^c^
Cytokine-cytokine receptor interaction	38	122	5.89E−09	3.29E−06
Glycine, serine and threonine metabolism	13	30	1.90E−04	1.90E−04
MAPK signaling pathway	40	231	4.26E−03	4.18E−04
Rheumatoid arthritis	17	53	4.57E−04	4.18E−04
Biosynthesis of amino acids	17	59	1.13E−03	1.35E−03
PI3K-Akt signaling pathway	44	249	1.54E−03	3.41E−03
Arrhythmogenic right ventricular cardiomyopathy (ARVC)	16	50	6.73E−04	3.41E−03
Prostate cancer	20	83	2.56E−03	3.41E−03
Mineral absorption	12	34	1.54E−03	3.41E−03
Fluid shear stress and atherosclerosis	26	112	8.46E−04	3.41E−03
Osteoclast differentiation	16	87	8.59E−02	1.64E−02
Transcriptional misregulation in cancer	24	128	2.31E−02	3.97E−02
Complement and coagulation cascades	9	35	6.20E−02	4.09E−02
Hypertrophic cardiomyopathy (HCM)	13	56	4.09E−02	4.09E−02

a countDE = number of differentially expressed genes in dataset; countAll = total number of genes in pathway.

b pORA_FDR = *P*-value based on overrepresentation alone, adjusted for false discovery rate.

c pOverall_FDR = *P*-value based on overrepresentation and degree of perturbation, adjusted for false discovery rate.

The Advaita iPathway software allowed us to additionally identify “upstream” hubs or mediators that regulate many of the genes in this dataset that may be downstream. We identified *P53* and *IL6* as statistically significant downregulated and upregulated hubs, respectively (Fig. 1D, Supplementary Fig. S1). These unbiased enrichment analyses support what has often been described in the literature as the ability of PM_2.5_ to induce DNA damage (often regulated by *P53*) (5, 7, 9) and cause increases in inflammatory cytokines (e.g. *IL6*) (17, 18). Finally, the Advaita software also allows one to examine potential upstream chemicals or toxicants that are often associated with these differentially expressed genes, and upstream chemicals include organophosphorus compounds, polyaromatic hydrocarbons, and metals such as copper, silver, nickel, and cadmium, which are all often found in PM_2.5_.

**Figure 2:**
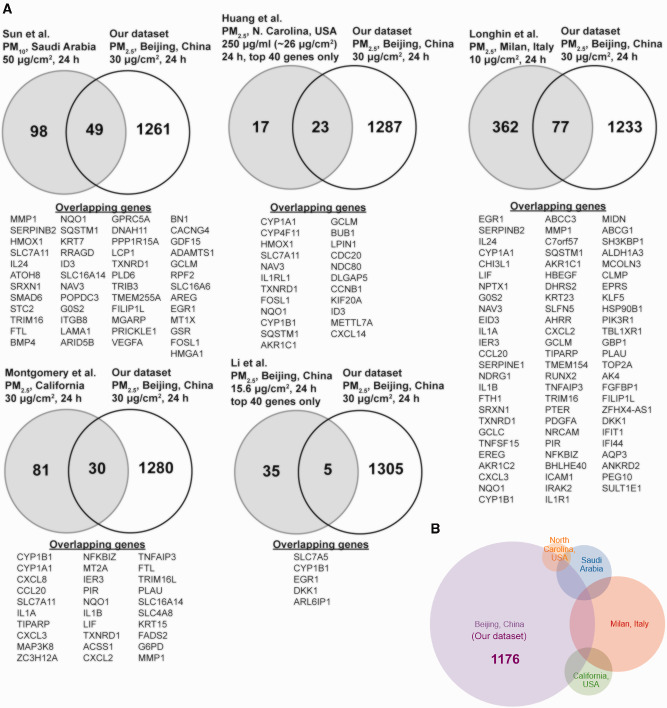
comparison of differentially expressed genes after 24-h treatment with PM_2.5_ (30 µg/cm^2^) with genes identified in other published datasets (10–13, 15). (**A**) The 1310 differentially expressed genes in our dataset were compared to differentially expressed genes found in other published datasets (10–13, 15), with the identity of the overlapping genes listed below. (**B**) Area-proportional Venn diagram of differentially expressed genes from different datasets with source of PM from different studies indicated.

We next used the GEO database and PubMed to identify other studies that also performed transcriptomic analysis in bronchial epithelial cells after PM exposure (Table 2). These studies utilized PM of varying size (2.5–10 µm) and from different locations [Saudi Arabia (15), China (11), United States (10, 13), Italy (12)], with treatments ranging from a concentration of 10–50 µg/cm^2^ for 24 h. Excluding studies where data were incomplete or not publicly available, we compared the differentially expressed genes found in our study with those identified in other datasets (Fig. 2). Several genes, including *NQO1*, *TXNRD1*, *CYP1A1*, and *CYP1B1*, were found to be differentially expressed after PM exposure in all studies. Our dataset, however, also identified a larger number of genes (1176 of them) that were not identified in any other published dataset (Supplementary Table S1). Although other studies (9, 16) identified enrichment of Kyoto Encyclopedia of Genes and Genomes (KEGG) pathways such as Cytokine–Cytokine Receptor Interaction, MAPK Signaling Pathway, and Rheumatoid Arthritis, that we also observed were enriched in our dataset, we found additional pathways such as Glycine Serine and Threonine Metabolism, Arrhythmogenic Right Ventricular Cardiomyopathy, Fluid Shear Stress and Atherosclerosis, and Complement and Coagulation Cascades that were enriched only in our dataset.

**Table 2:** a list of comparative gene expression studies investigating the effects of PM on bronchial epithelial cells

Study	Cell type	PM source	PM type	Concentration and duration	Method	# Differentially expressed genes	GEO ID
Nakayama *et al*. (14)	HBE cells	Fresno, California Summer 2006, Winter 2007	Ambient and wildfire PM_2.5_	10 µg/ml, 3 h exposure	Affymetrix Human U133A 2.0 microarray	127	GSE18593
Sun *et al*. (15)	BEAS-2B	Saudi Arabia	PM_10_	50 µg/cm^2^, 24 h	Affymetrix 1.0 array	147	GSE38172
Huang *et al*. (10)	Primary human airway epithelium	Chapel Hill, NC, October 2002	Coarse, fine, ultrafine PM	250 µg/ml (∼26 µg/cm^2^), 24 h	Affymetrix Human U133A microarray	302^a^	GSE7010
Montgomery *et al*. (13)	Nasal airway epithelium in air–liquid interface	Urban California (cities of Bakersfield, Sacramento, and Yuba City), 2011; NIST 2786	PM_2.5_ - water soluble and organic extracts	Organic extract: up to 4.5 µg/cm^2^, 24 h; NIST: 30 µg/cm^2^, 24 h	RNA-Seq	11, 124, and 1296 from low, moderate, and high dose organic extract; 111 from NIST	GSE144770
Li *et al*. (11)	BEAS-2B	Beijing, China, December 2015	PM_2.5_	50 µg/ml (∼15.625 µg/cm^2^), 24 h	Affymetrix Human Transcriptome Array 2.0	1636^b^	GSE93329
Longhin *et al*. (12)	BEAS-2B	Milan, Italy	Summer PM_10_, winter PM_2.5_	10 µg/cm^2^, 24 h	Affymetrix Human U133 Plus 2 microarray	441 for winter PM_2.5_; 542 for summer PM_10_	ArrayExpress E-MTAB-3630
Zhou *et al*. (16)	16HBE cells	Beijing, China, January 2013	PM_2.5_	25 µg/cm^2^, 24 h	RNA-Seq	539	Unavailable
Ding *et al*. (9)	HBE cells	Wuhan, China, March 2012	PM_2.5_	200 and 500 µg/ml, 24 h	Microarray	970 with 200 µg/ml; 492 with 500 µg/ml	Unavailable

NIST, National Institute of Standards and Technology; HBE, Human bronchial epithelial.

a Only top 40 available for download and comparison.

b Only top 40 used for comparison.

In all prior transcriptomic studies, the lowest concentration of PM_2.5_ used for transcriptomic analysis was 10 µg/cm^2^ PM_2.5_; we and others, however, have shown that concentrations lower than 10 µg/cm^2^ were sufficient to alter the expression of some genes, and in some instances, altered the expression of genes that were not affected by higher concentrations of PM_2.5_ (20). We thus performed RNA-Seq on cells treated 24 h with 1 µg/cm^2^ of PM_2.5_. The number of genes altered after exposure to a low-concentration of PM_2.5_ was considerably less compared to the number of differentially expressed genes after treatment with higher concentrations of PM_2.5_. In fact, of the 1310 genes that were differentially expressed after exposure to 30 µg/cm^2^ of PM_2.5_, only 154 (12%) showed differential expression (based on log fold-change ≥ 0.6) after low-concentration PM_2.5_. Treatment with lower concentrations of PM_2.5_ often resulted in gene expression changes that were in the same direction as higher concentrations, but with a lower magnitude of effect (Supplementary Table S3). However, some genes such as *TIE1*, *IL1RL1*, *DUSP5*, *CXCL8*, *ADTRP*, *LEF1*, *GPR3*, *FOS*, *PTX3*, *SERPINE1*, *FOSL1*, *AQP3*, *PADI2*, and *BMF* exhibited opposing directions of expression after either low or high concentrations of PM_2.5_. Using a less stringent statistical model based on likelihood ratios (which has traditionally been used in many transcriptomic studies) rather than F-tests, we identified 40 genes (using the same false discovery rate < 0.05) that were differentially expressed to a statistically significant degree after low-concentration exposure to PM_2.5_ (Table 3). Certain genes such as *FYB*, *RCAN2*, *WNT9A*, and *CTGF* were affected by low-concentration but not high concentration of PM_2.5_, suggesting that expression of several genes may be uniquely sensitive to these lower concentrations.

**Table 3:** differentially expressed genes in cells treated with PM2.5 vs. Control, 24 h

		Effect of low-dose (1 µg/cm^2^) PM_2.5_			Effect of high dose (30 µg/cm^2^) PM_2.5_	
Gene symbol	Entrez ID	log fold-change	Adjusted *P*-value	Likelihood ratio	log fold-change	Adjusted *P*-value
CYP1A1	1543	6.88	2.10E−24	122.99	4.34	2.89E−04
FYB	2533	4.35	3.65E−02	15.66		NS
C2orf54	79919	2.49	3.46E−02	15.83		NS
AHRR	57491	2.43	3.46E−23	116.05	1.52	3.18E−04
RCAN2	10231	2.38	4.14E−03	20.85		NS
NPTX1	4884	2.27	4.49E−02	14.88	3.14	3.62E−03
CYP1B1	1545	2.06	3.48E−23	115.24	1.90	3.06E−05
NKD2	85409	2.05	6.50E−05	29.99	1.67	5.28E−03
ITGA11	22801	1.90	6.89E−06	34.55	1.90	1.15E−03
IL24	11009	1.75	5.18E−03	20.31	4.36	1.86E−05
NMRAL2P	344887	1.62	4.49E−02	14.84	5.23	7.95E−06
TMEM229B	161145	1.49	6.87E−03	19.58		NS
RUNX2	860	1.46	1.04E−02	18.71	1.39	1.44E−02
LINC00886	730091	1.44	1.76E−06	37.70		NS
SLC7A5	8140	1.35	3.11E−16	82.97	1.13	1.56E−04
ARTN	9048	1.35	2.11E−04	27.20		NS
TIPARP	25976	1.34	1.91E−02	17.46	1.44	9.37E−03
VIPR1	7433	1.22	4.49E−02	15.00		NS
CADM1	23705	1.22	2.41E−02	16.58		NS
TRIM16L	147166	1.16	1.20E−04	28.62	2.44	1.69E−05
LINC00511	400619	1.16	2.61E−03	21.84		NS
RP11-66B24.7	NA	0.98	4.16E−06	35.77		NS
NQO1	1728	0.92	2.99E−04	26.38	1.95	1.93E−05
WNT9A	7483	0.92	6.87E−03	19.59		NS
SLC45A4	57210	0.75	4.49E−02	14.84		NS
CLMP	79827	0.69	1.99E−02	17.30	0.71	6.34E−03
TSC22D1	8848	−0.58	2.02E−02	17.15		NS
SEMA3A	10371	−0.64	3.79E−02	15.53	−1.66	1.69E−05
RP3-331H24.7	NA	−0.75	3.81E−02	15.41		NS
PDE4D	5144	−0.78	4.49E−02	14.94	−0.69	2.58E−02
DUSP1	1843	−0.78	3.81E−02	15.41	−1.06	2.39E−03
PEG10	23089	−0.81	4.53E−02	14.77	−3.06	7.69E−06
LOX	4015	−0.85	1.21E−04	28.43	−1.81	1.05E−05
IFI44	10561	−0.94	1.44E−03	23.22	−1.68	7.96E−05
EFEMP1	2202	−1.05	6.25E−07	40.03	−2.21	7.69E−06
PCDH18	54510	−1.09	1.92E−03	22.54	−2.33	3.58E−05
SMOC1	64093	−1.40	4.21E−07	41.15	−1.71	1.91E−04
SLITRK6	84189	−1.88	2.16E−02	16.92	−2.59	3.39E−03
CTGF	1490	−2.43	2.19E−02	16.82		NS
RGS4	5999	−3.79	2.02E−02	17.12	−2.61	4.03E−02

NS = not significant.

### Transcriptomic Changes in BEAS-2B Cells after Repeated Exposures for Seven Days

**Figure 3:**
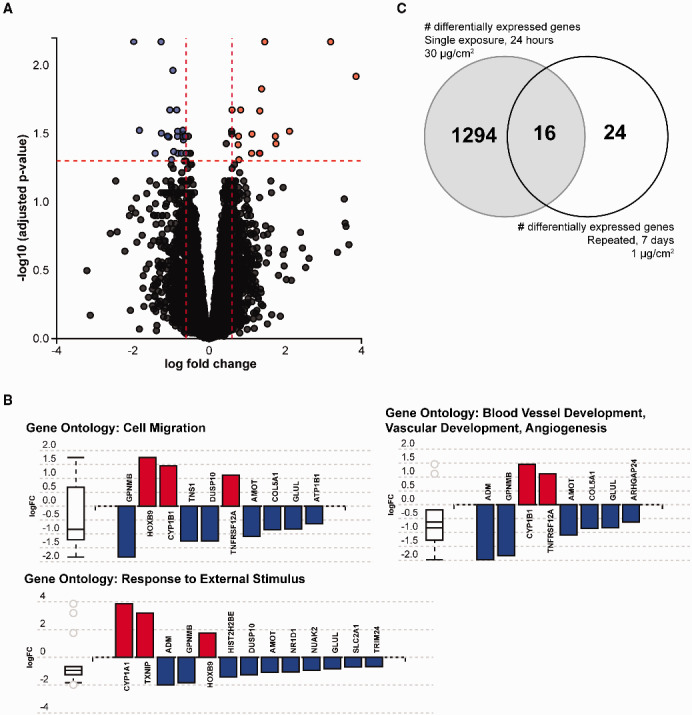
differential expression of genes after repeated, low-concentration exposure with PM_2.5_. BEAS-2B cells were treated with a fresh dose of 1 µg/cm^2^ of PM_2.5_ on a daily basis for six consecutive days. Cells were washed each day between consecutive treatments. At day seven, cells were isolated for RNA analysis and gene expression changes by fold-change and adjusted *P*-value are shown in the volcano plot (**A**). (**B**) Enrichment analyses identified specific gene ontologies that were enriched among the differentially expressed genes after repeated low-concentration exposure. (**C**) Comparison of differentially expressed genes after a single 24-h exposure with 30 µg/cm^2^ of PM_2.5_ and differentially expressed genes after repeated exposure to low-concentration PM_2.5_ identified a number of overlapping and non-overlapping genes.

Experimental models that simulate chronic daily exposure have increasing relevance to humans who are exposed to low levels of pollution on an everyday basis. We have observed that expression of some genes is not altered when cells are exposed to a single dose of PM_2.5_, but increased when exposure is repeated daily (20). To examine the potential for transcriptomic changes that occur with this model, cells were treated every day with a low-concentration of PM_2.5_ (1 µg/cm^2^) for one week. To prevent the accumulation of additive doses, cells were washed each day between treatments. We identified 40 genes (16 upregulated and 24 downregulated) that were differentially expressed in cells treated with chronic daily exposure to PM_2.5_ compared to control (Fig. 3A and Table 4). Analysis of gene ontology identified biological processes such as blood vessel development, cell migration, and response to external stimulus to be enriched in this group. The relative expression of genes within each gene ontology group is shown in Fig. 3B. Interestingly, of these genes affected by chronic daily exposure, only 4 genes, *CYP1A1*, *CYP1B1*, *AHRR*, and *LINC00886*, were also shown to be affected after a single 24-h exposure with the same concentration of PM_2.5_ (1 µg/cm^2^). The lack of many more genes in common between single (Table 3) and repeated (Table 4) treatments with low-concentrations of PM_2.5_ emphasize the importance of considering duration of treatment on gene expression. Because there were more genes that were affected by a single 24-h exposure using higher concentrations (30 µg/cm^2^) of PM_2.5_, we also compared the genes that were differentially expressed after a single exposure with high concentration of PM_2.5_ with those that were differentially expressed after repeated, low-concentration of PM_2.5_ treatment. Only 16 of the 40 (40%) genes that were differentially expressed after chronic, repeated exposure to low-concentrations of PM_2.5_ were also affected when cells were treated with a single, 24-h exposure of high-concentration PM_2.5_ (Table 4). These data highlight the differing responses of bronchial epithelial cells when exposed to either single or repeated doses of PM_2.5_.

**Table 4:** differentially expressed genes in cells after treatment with PM_2.5_ (1 µg/cm^2^), repeated 7 days

		Effect of PM_2.5_ 1 µg/cm^2^, 7 days			Effect of PM_2.5_ 30 µg/cm^2^, 24 h	
Gene symbol	Entrez ID	Log fold-change	Adjusted *P*-value	F value	Log fold-change	Adjusted *P*-value
CYP1A1	1543	3.85	1.20E−02	54.60	4.34	2.89E−04
TXNIP	10628	3.19	6.72E−03	65.45		NS
ARRDC4	91947	2.11	3.04E−02	36.79		NS
HOXB9	3219	1.75	3.74E−02	30.27	2.98	1.57E−04
DLG1-AS1	100507086	1.74	3.30E−02	32.25		NS
CYP1B1	1545	1.46	6.72E−03	71.06	1.90	3.06E−05
AHRR	57491	1.38	1.49E−02	50.47	1.52	3.18E−04
LINC00886	730091	1.33	2.15E−02	41.12		NS
PRSS21	10942	1.33	4.40E−02	27.75	0.75	4.45E−02
KCNAB3	9196	1.32	4.40E−02	28.46		NS
LINC00680	106660612	1.13	3.18E−02	34.58		NS
TNFRSF12A	51330	1.12	4.40E−02	27.96	1.44	7.33E−04
RPS6KL1	83694	0.83	2.12E−02	41.85		NS
TMEM147-AS1	100506469	0.78	4.90E−02	26.76		NS
LINC00909	400657	0.77	3.30E−02	32.37		NS
FAM173B	134145	0.76	3.81E−02	29.96		NS
CSTF3	1479	0.60	2.12E−02	42.25		NS
PLEKHA2	59339	−0.61	4.40E−02	27.64	−0.86	3.91E−04
ARHGAP24	83478	−0.62	3.30E−02	33.62	−1.10	4.83E−05
ATP1B1	481	−0.64	3.30E−02	32.36	−0.65	2.12E−03
TRIM24	8805	−0.67	3.51E−02	31.00		NS
TBC1D2	55357	−0.68	3.34E−02	31.92		NS
SLC2A1	6513	−0.69	2.99E−02	37.56		NS
ALDH6A1	4329	−0.69	3.18E−02	34.49		NS
ENDOD1	23052	−0.72	4.40E−02	27.64		NS
COL4A4	1286	−0.82	4.40E−02	27.98	−1.46	8.73E−05
GLUL	2752	−0.82	3.04E−02	36.43	−0.82	1.68E−03
DIAPH2	1730	−0.83	3.30E−02	32.20		NS
COL5A1	1289	−0.85	2.12E−02	43.54	−0.96	3.91E−04
KRT5	3852	−0.93	4.27E−02	29.18	−2.46	7.95E−06
NUAK2	81788	−0.95	1.09E−02	56.70		NS
TP63	8626	−0.99	4.90E−02	27.10		NS
ARL4C	10123	−1.03	2.12E−02	42.85		NS
NR1D1	9572	−1.05	3.35E−02	31.76	0.67	3.57E−02
AMOT	154796	−1.09	3.30E−02	32.61	−1.05	3.36E−03
DUSP10	11221	−1.25	3.14E−02	35.57		NS
TNS1	7145	−1.26	6.72E−03	64.39	−1.79	3.01E−05
HIST2H2BE	8349	−1.41	4.40E−02	28.29		NS
GPNMB	10457	−1.83	2.99E−02	38.36		NS
ADM	133	−1.98	6.72E−03	72.64		NS

NS = Not significant.

### Single Exposure to PM_2.5_ Induce DNA Methylomic Changes in BEAS-2B Cells

Exposure to air pollution, and PM_2.5_ specifically, has been well-recognized to cause changes in DNA methylation as measured *in vivo* (26–28) and in *in vitro* models (38, 39). These DNA methylation changes are capable of altering the gene expression and phenotype of cells and organisms. We performed eRRBS to first examine the DNA methylomic changes in BEAS-2B cells after 24 h treatment with PM_2.5_. Overall coverage was similar between control and treated samples and among all replicates. An average of 5 million CpG sites was covered by eRRBS per sample. The distribution of coverage, annotated to CpG islands, shores, shelves, or intergenic regions is shown in Supplementary Fig. S2.

**Figure 4:**
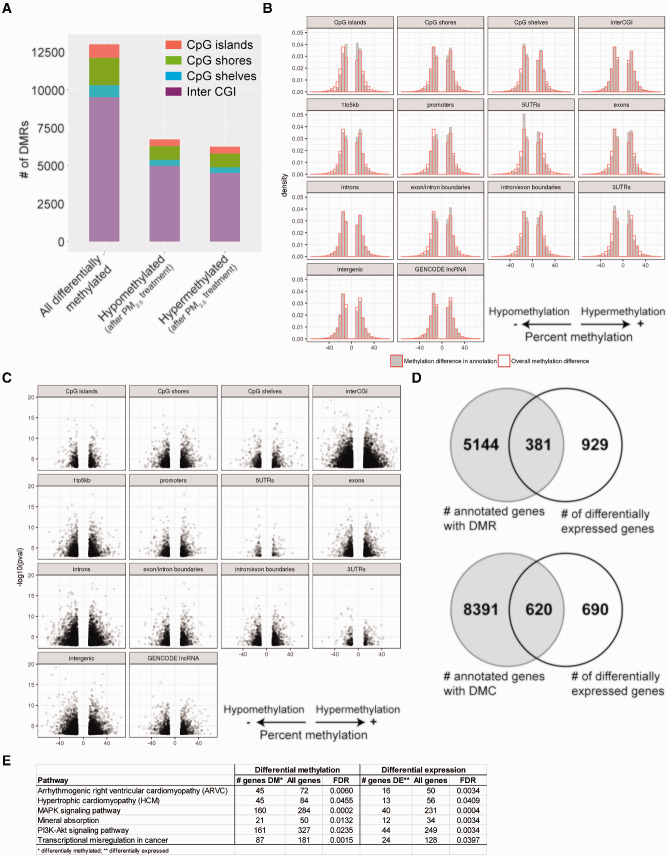
DNA methylation changes after 24-h treatment with 30 µg/cm^2^ of PM_2.5_. (**A**) Graphical representation of the number of differentially methylated regions (DMRs, defined by >10% difference in methylation and adjusted *P*-value < 0.05), broken down by location (CpG islands vs. shores vs. shelves) and relative methylation change (hyper- vs. hypo-) after PM_2.5_ treatment. (**B**) Density of differentially methylated regions mapped by gene annotation relative to overall difference in methylation observed. (**C**) Volcano plot of differentially methylated regions with degree and direction of DNA methylation change plotted against adjusted *P*-value. (**D**) Venn diagram of genes with differentially methylated regions (DMRs) or at least one differentially methylated CpG locus (DMCs) compared to genes that were differentially expressed. (**E**) Pathway analysis of genes that were both differentially expressed and differentially methylated after single-dose PM_2.5_ exposure identified specific statistically enriched pathways common to both groups.

Cells treated with 30 µg/cm^2^ of PM_2.5_ demonstrated widespread changes in DNA methylation throughout the genome (Fig. 4). There were 73 648 differentially methylated CpGs (DMCs) and 12 810 differentially methylated CpG regions (DMRs) between treatment and control (Supplementary Table S4). Among the DMRs, there were slightly more regions that were hypomethylated than hypermethylated. Hypermethylated regions were found disproportionately more among CpG islands and shores, whereas hypomethylated changes were found in greater proportion among intergenic regions (Fig. 4A and B).

A breakdown of methylation changes relative to regions within genes is shown in Fig. 4B. Although PM_2.5_ caused a slight increase in overall hypomethylation across the genome, a higher proportion of hypermethylation was found in the 1–5 kb upstream regions, promoters, and exon/intron boundaries. Conversely, a greater proportion of hypomethylation was found in 5′-untranslated regions. Volcano plots of differentially methylated regions and their annotations to gene region are shown in Fig. 4C. Pathway analysis among the genes that were differentially methylated identified a variety of different enriched pathways (Supplementary Table S5). Some of these were the same pathways as that observed among differentially expressed genes, such as MAPK Signaling Pathway, Pathways in Cancer, and Arrhythmogenic Right Ventricular Cardiomyopathy. However, there were many other additional pathways that were enriched among the differentially methylated dataset.

We next correlated changes in DNA methylation with gene transcription. When all of DMCs were taken into account, there were 9011 genes that had least one differentially methylated CpG locus. Likewise, the 12 810 DMRs annotated to 5525 gene IDs. Of the 1310 differentially expressed genes, 381 were found to have DMRs and nearly half (620/1310) were found to have at least one differentially methylated cytosine (DMC) (Fig. 4D). Supplementary Table S6 lists those genes with differential expression and at least one differentially methylated region (DMR). Among the genes with both differential expression and differential methylation, pathway analysis identified enrichment in the specific pathways Hypertrophic Cardiomyopathy, MAPK Signaling Pathway, PI3K–Akt Signaling Pathway, and Transcriptional Misregulation in Cancer (Fig. 4E).

DNA methylomic analysis was also performed on BEAS-2B cells treated with a low-concentration of PM_2.5_ (1 µg/cm^2^) for 24 h. Although the number of genes that were differentially expressed after exposure to PM_2.5_ was less in the low-concentration treatment group compared to the high-concentration group, many of the genes that were differentially expressed also demonstrated differential methylation (Table 5).

**Table 5:** genes differentially expressed and methylated in cells after low-dose (1 µg/cm^2^) PM_2.5_ treatment

Gene symbol	Entrez ID	log fold-change	Adjusted *P*-value	Chromosome	DMR start	DMR end	Condition hypermethylated	Methylation in PM_2.5_ (%)	Methylation in control (%)	Methylation difference (%)	Adjusted *P*-value	Annotation type
After 24 h, single exposure												
PDE4D	5144	−0.78	4.49E−02	chr5	59658501	59658550	PM24hours1dose	61.33	30.23	31.10	1.37E−02	intron : 289214
PDE4D	5144	−0.78	4.49E−02	chr5	59681801	59681850	PM24hours1dose	85.57	72.28	13.29	1.12E−02	intron : 289214
PDE4D	5144	−0.78	4.49E−02	chr5	59736351	59736400	Control24hours	57.09	77.17	−20.08	4.79E−02	intron : 289214
WNT9A	7483	0.92	6.87E−03	chr1	227941851	227941900	Control24hours	87.30	100.00	−12.70	7.80E−03	intron : 86681
CADM1	23705	1.22	2.41E−02	chr11	115462251	115462300	Control24hours	86.96	99.18	−12.22	7.30E−03	intron : 548669
CADM1	23705	1.22	2.41E−02	chr11	115506251	115506300	Control24hours	45.63	75.95	−30.32	3.93E−02	1to5kb : 101713
CADM1	23705	1.22	2.41E−02	chr11	115509201	115509250	PM24hours1dose	48.53	35.06	13.47	4.89E−02	1to5kb : 101713
AHRR	57491	2.43	3.46E−23	chr5	315101	315150	Control24hours	78.48	98.26	−19.78	5.59E−03	intron : 262115
AHRR	57491	2.43	3.46E−23	chr5	434801	434850	Control24hours	61.70	72.25	−10.54	2.04E−02	exon : 309787
SMOC1	64093	−1.40	4.21E−07	chr14	69854551	69854600	PM24hours1dose	25.47	12.90	12.57	5.09E−03	intron : 634135
SMOC1	64093	−1.40	4.21E−07	chr14	69900401	69900450	Control24hours	76.51	90.32	−13.81	3.83E−02	intron : 634137
C2orf54	79919	2.49	3.46E−02	chr2	240891601	240891650	Control24hours	40.85	60.77	−19.93	1.38E−07	exon : 192406
TMEM229B	161145	1.49	6.87E−03	chr14	67453201	67453250	Control24hours	31.35	48.71	−17.36	4.48E−02	intron : 651452
LINC00511	400619	1.16	2.61E−03	chr17	72346501	72346550	Control24hours	78.03	92.77	−14.74	3.43E−04	lincRNA : 10882
LINC00511	400619	1.16	2.61E−03	chr17	72364501	72364550	Control24hours	86.12	97.01	−10.89	2.44E−02	lincRNA : 10882
After 7 days of repeated daily exposure												
TNS1	7145	−1.26	6.72E−03	chr2	217818651	217818700	Control7days	72.95	86.76	−13.82	4.77E−02	exon : 187264
TNS1	7145	−1.26	6.72E−03	chr2	217928301	217928350	PM7days	93.33	60.71	32.62	3.75E−02	intron : 158857
LINC00886	730091	1.33	2.15E−02	chr3	156764551	156764600	Control7days	77.35	89.50	−12.15	8.85E−03	lincRNA : 2738
AHRR	57491	1.38	1.49E−02	chr5	346651	346700	Control7days	79.62	90.54	−10.92	2.89E−02	intron : 262116
COL5A1	1289	−0.85	2.12E−02	chr9	134711101	134711150	PM7days	95.74	83.73	12.01	3.66E−03	intron : 442076
COL5A1	1289	−0.85	2.12E−02	chr9	134793651	134793700	Control7days	57.68	68.33	−10.66	4.53E−02	intron : 442104
COL5A1	1289	−0.85	2.12E−02	chr9	134842301	134842350	Control7days	55.37	67.23	−11.86	4.99E−02	exon : 524058

### DNA Methylomic Changes in Cells Exposed to Daily Repeated Exposures

**Figure 5:**
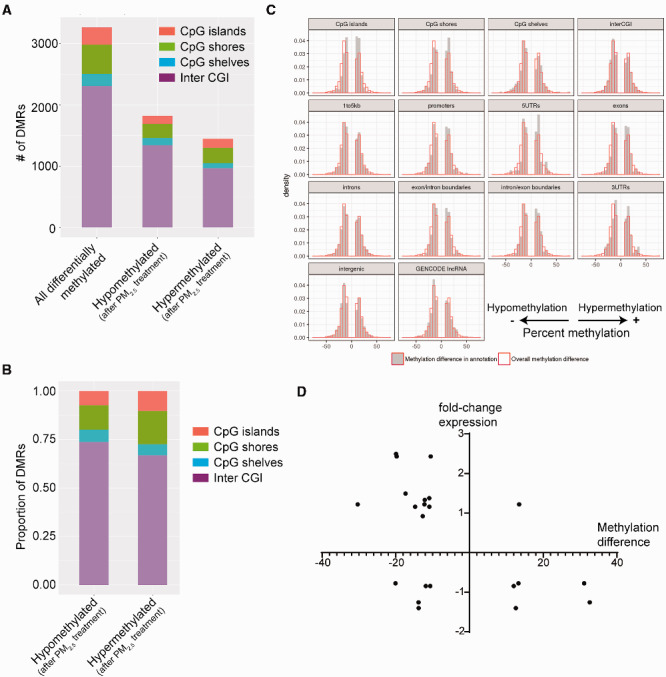
DNA methylation changes after repeated, daily exposure of BEAS-2B to 1 µg/cm^2^ of PM_2.5_. (**A**) Graphical representation of the number of DMRs (defined by >10% difference in methylation and adjusted *P*-value < 0.05), broken down by location (CpG islands vs. shores vs. shelves) and relative methylation change (hyper- vs. hypo-) after PM_2.5_ treatment. (**B**) Graphical representation of hyper- and hypomethylated DMRs broken down by location (CpG islands vs. shores vs. shelves) expressed as a proportion of total hyper- or hypomethylated DMRs. (**C**) Density of differentially methylated regions mapped by gene annotation relative to overall difference in methylation observed. (**D**) Comparison of the change in DNA methylation (based on average methylation difference in DMR) with the fold-change in gene expression of the differentially expressed genes identified in single 24-h low-concentration and repeated low-concentration PM_2.5_ exposure protocols.

Cells exposed to PM_2.5_ with repeated doses may develop different DNA methylation changes over time. We thus performed eRRBS on our seven-day exposure protocol. There were 18 641 DMCs and 3213 DMRs in cells that were treated daily for seven days with low-concentration PM_2.5_ compared to control. Similar to what we observed with single-dose short-term exposure conditions, treatment of epithelial cells with repeated doses of PM_2.5_ resulted in a greater number of hypomethylated than hypermethylated DMRs, and this difference was even more pronounced than compared to our short-term treatment protocol (Fig. 5A). Although repeated treatment with PM_2.5_ caused a greater degree of hypomethylation across the genome, a greater proportion of the hypermethylated regions were found in CpG islands and shores, with hypomethylated regions found more predominantly in intergenic regions (Fig. 5A–C). DNA hypermethylation was also found disproportionately more in promoters, exons, and exon/intron boundaries, whereas DNA hypomethylation was found more in 3′- and 5′-untranslated regions. This pattern was similar to what we had observed in our 24 h experimental protocols.

Overall, the 3213 DMRs that resulted after chronic, repeated exposure to PM_2.5_ were annotated to 1844 genes. Nearly half of these genes were different from those that were differentially methylated after single exposure. The genes that were differentially methylated after chronic, repeated exposure to PM_2.5_ also not surprisingly demonstrated enrichment of pathways that were different from the pathways identified from differentially methylated genes after single PM_2.5_ exposure.

To determine whether changes in DNA methylation influences gene expression, we examined the DNA methylation levels of the 40 genes that were differentially expressed by PM_2.5_ after repeated exposure. Of the 40 genes, *AHRR*, *COL5A1*, *TNS1*, and *LINC00886* were identified as both differentially expressed and methylated (Table 5). We next correlated the degree and direction of DNA methylation changes with differential gene expression among cells treated with low-concentration of PM_2.5_, either for 24 h or repeatedly for seven days. Overall, genes that were hypomethylated were more often associated with increased expression and genes that were hypermethylated were associated with diminished expression (Fig. 5D).

**Figure 6:**
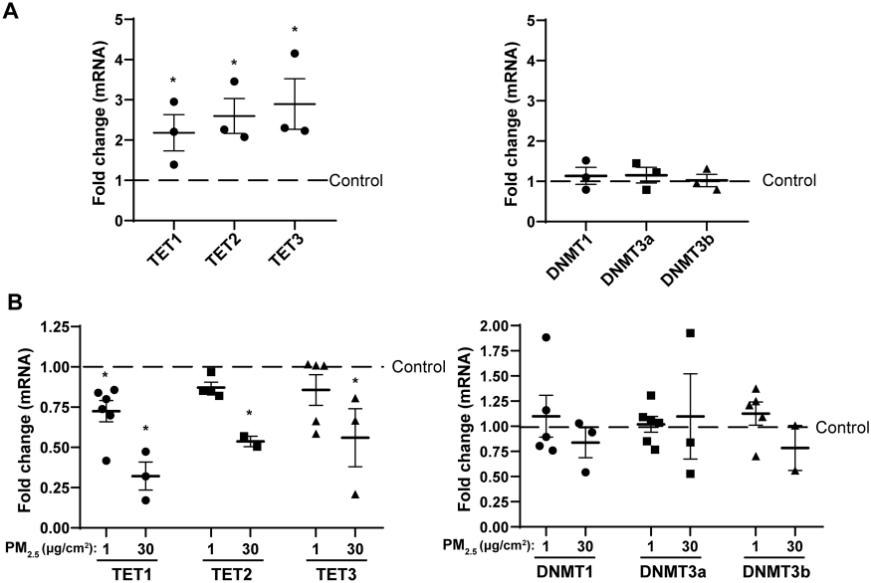
expression of ten–eleven translocation (TET) and DNA methyltransferase (DNMT) enzymes after PM_2.5_ treatment. (**A**) Expression of TET1-3 and DNMT1, -3a, and -3 b in cells after daily, repeated exposure to 1 µg/cm^2^ of PM_2.5_ for seven days, relative to untreated control. (**B**) Relative expression of TET1-3 and DNMT1, -3a, and -3b after single, 24-h treatment with PM_2.5_. *P < 0.05, one-way ANOVA with Tukey’s multiple comparisons post-test.

Since treatment with PM_2.5_ resulted in an overall greater degree of DNA hypomethylation across the genome, we next examined expression of DNA methyltransferases (DNMTs) and ten–eleven translocation (TET) enzymes, which promote hydroxymethylation and eventual demethylation, in our experimental conditions. Single treatment with PM_2.5_ for 24 h had no effect on DNA methyltransferase (DNMT) expression and caused a decrease in TET expression, but daily treatment with low-concentration of PM_2.5_ for seven days resulted in an increase in expression of TET1, TET2, and TET3 (Fig. 6).

## Discussion

In this study, we describe the transcriptomic and DNA methylomic changes that occur in bronchial epithelial cells after 24 h of either high (30 µg/cm^2^) or low (1 µg/cm^2^) concentration treatment with PM_2.5_ from Beijing, China. We also examine the transcriptomic and DNA methylomic changes that occur in cells after repeated exposure to 1 µg/cm^2^ of PM_2.5_ every day for seven days. Overall, we found widespread changes in the transcriptomic and DNA methylomic patterns of bronchial epithelial cells after PM_2.5_ treatment. These changes differed depending on the concentration and duration of exposure. Treatment with 30 µg/cm^2^ of PM_2.5_ altered the expression of a large number of genes, many of which included cytokines and which were enriched in MAPK, PI3K-Akt, IL6, and P53 pathways. Although a lower number of genes were differentially expressed after treatment with 1 µg/cm^2^ of PM_2.5_ compared to 30 µg/cm^2^ of PM_2.5_, repeated exposure to low-concentrations of PM_2.5_ over several days resulted in the differential expression of many genes that were interestingly, different from those observed after 24 h of PM_2.5_ treatment. Treatment with PM_2.5_ resulted in extensive changes in DNA methylation, and many of these DNA methylation changes were found in genes that also demonstrated differential expression. Overall, there were more hypomethylated changes compared to hypermethylated changes after PM_2.5_ exposure, especially in cells treated repeated for seven days with PM_2.5_. More hypermethylated changes were noted in CpG islands and shores while hypomethylated changes were predominantly in intergenic regions. Together, these findings demonstrate the ability of PM_2.5_ to induce widespread changes in gene expression and DNA methylation under different concentrations and duration of PM_2.5_ exposure. The extent of these changes provides insight into how PM_2.5_ affects airway epithelial cell biology, inflammatory responses, and ultimately, disease.

Treatment with high concentrations of PM_2.5_ (30 µg/cm^2^) induced upregulation of a number of genes that were associated with toxicological responses to PM_2.5_ and that have been described in the literature. We observed upregulation of *CYP1A1*, *CYP1B1*, and *AHRR*, which is consistent with the polyaromatic hydrocarbon content of PM_2.5_ (7, 35). PM_2.5_ is also known to induce oxidative stress, commonly through the transcription factor NRF2 (36, 37). NRF2 binds to the anti-oxidant response element and can activate genes such as *HMOX1*, *NQO1*, *GCLM*, *SQSTM1*, and *FTL*, which were all increased in the dataset. Perhaps not surprising were also increased expression of *MDM2* and *CDKN1A*, which were expected to occur with PM-associated DNA damage (5), or increased expression of cytokines such as *IL6*, *IL1B*, *TSLP*, *CXCL2*, and *CXCL3*, which have all been demonstrated to be upregulated in other studies (11, 12, 16). Metals such as silver, copper, cadmium, and nickel, which are present in PM_2.5_, were not surprisingly identified in our unbiased pathway analyses as enriched upstream chemical regulators. These findings emphasize the validity and reproducibility of our results in the context of expected changes reported in the literature.

Our RNA-Seq data, however, also identified a much larger number of genes, 1310, that were differentially expressed after treatment with 30 µg/cm^2^ of PM_2.5_, which demonstrates the broad extent by which PM_2.5_ is capable of altering gene transcription in airway epithelial cells. Even when employing a more stringent cutoff for fold-change, at least 709 of these genes had at least greater than 2-fold-change in expression. We identified a much larger number of differentially expressed genes than that reported in most other studies (9–16), which may be attributed to the fact that we employed RNA-Seq, which is a more sensitive technique and provides a more comprehensive and broader analysis of transcriptomic changes than microarray analysis. By identifying a larger number of genes that were not previously noted in prior studies of PM_2.5_, we could discover additional pathways based on gene ontology that are altered by PM_2.5_, and identify novel genes that may provide additional insights into the effects of PM_2.5_ on epithelial cell biology. Certain genes such as *CYP1A1*, *CYP1B1*, *NQO1*, and *TXNRD1* were differentially expressed in all studies (9–16) including ours, demonstrating common, universal effects of PM_2.5_, even when they are from different regions of the world. Likewise, pathway analysis identified many gene ontology pathways that were commonly activated among different studies of PM_2.5_, including Cytokine–Cytokine Receptor Interaction and PI3K–Akt and MAPK Signaling. However, we also identified Glycine Serine and Threonine Metabolism, Arrhythmogenic Right Ventricular Cardiomyopathy, Fluid Shear Stress and Atherosclerosis, and Complement and Coagulation Cascades as potentially important pathways that were enriched only in our dataset.

Unique to our study was the fact that we examined gene expression changes with concentrations of 1 µg/cm^2^ of PM_2.5_, which was lower than that used in other transcriptomic studies. The rationale to study the effects of PM_2.5_ at this low-concentration was based on the fact that we had observed differential gene expression changes occurring with this concentration (sometimes even without changes at higher concentrations) (20), and low-concentrations of PM_2.5_ have been shown to exert important effects in other studies (17–19). We found fewer genes that were differentially expressed after treatment with a low-concentration of PM_2.5_ compared to higher concentrations, and the magnitude of effect was often less after lower concentrations of PM_2.5_ compared to higher concentrations. There were, however, certain genes that were affected by low-concentration PM_2.5_ treatment that were not observed after high-concentration treatment, and this illustrates how low levels of PM_2.5_ exposure cause changes that are distinct from higher concentrations of PM_2.5_ exposure. This is consistent with what we had observed in focused studies of select genes (20), though the mechanism for this is unclear. One potential hypothesis is that low-concentrations of PM_2.5_ might activate transcription factors that are opposed by other transcription factors that are activated at only higher concentrations of PM_2.5_. Additionally, signaling pathways activated by PM_2.5_ at low concentrations might be inhibited by pathways activated by only high concentrations, such as when anti-oxidant pathways become activated to counter the effects of oxidative stress. Finally, different concentrations of PM_2.5_ may activate signaling pathways with different kinetics and some genes may be activated by only certain pathways and not others. Ultimately, there were not enough genes activated by only low-concentration but not high-concentration PM_2.5_ that allowed us to perform rigorous pathway analyses, but future studies with increased sample size may enhance the statistical power to identify even more genes uniquely affected by low-concentration of PM_2.5_.

Studying the effects of repeated treatment with low-concentrations of PM_2.5_ over time has the potential to further model real-world exposure, since much of the world population are exposed to low levels of PM_2.5_ on a chronic, daily basis. Here, we found that repeated exposures induced gene expression changes that are distinct from what was observed after a single, 24-h exposure protocol. This was true even when comparing the effects of chronic exposure to the 24-h changes associated with higher concentrations of PM_2.5_, indicating that the effects of repeated exposure cannot be explained merely by the arithmetic total of accumulated PM_2.5_. This differential effect of PM_2.5_ between single and chronic, repeated treatment protocols has been described in other studies as well, in the context of epithelial mesenchymal transition and histone modifications (17–19). Along with these other studies, our data emphasize both the need and importance of utilizing models of repeated treatment to assess the effects of chronic exposure to PM_2.5_ and highlights the profound changes, both transcriptomic and epigenetic, that occur when cells are exposed to PM_2.5_ over time. The mechanisms for this may vary but could include adaptive changes in cells that are chronically exposed to PM_2.5_, as often observed when anti-oxidant signals become upregulated in response to oxidant damage. Epigenetic modifications may account for some of these adaptive changes, and many studies have examined how air pollutants affect DNA methylation in humans, not just acutely, but over the life course of an individual (26, 28, 40). One study showed that exposure of BEAS-2B cells to biomass from a power plant for five weeks induced widespread DNA methylation and gene expression differences (38). Exactly how epigenetic and gene expression changes continue to evolve over time remains to be determined.

DNA methylation changes represent an important epigenetic mechanism that affects gene expression, often in a persistent manner, and that contributes to the development of many diseases including cancer (21) and asthma (23). Both *in vivo* and *in vitro* studies have shown that air pollution affects DNA methylation patterns in a variety of cell types (26–28). Here, we utilized eRRBS to examine the DNA methylomic changes in bronchial epithelial cells after PM_2.5_ treatment and found that PM_2.5_ induced widespread DNA methylomic changes, many of which correlated with changes in gene expression. These changes occurred with both short-term and long-term PM_2.5_ exposures, though the genes that were affected differed between exposure protocols. As compared to other studies in air pollution that utilized microarrays for DNA methylation analysis (38, 39), which are biased toward pre-determined CpG sites, we employed eRRBS, which assays a more diverse region of genes and broader coverage. Our dataset thus has the potential to provide richer insights into the epigenetic changes induced by PM_2.5_ and the mechanisms by which PM_2.5_ affects individual gene expression. However, eRRBS has its own limitations, and in our study, only 47.6% of Illumina 450 K array and 37.3% of Illumina EPIC array (850 K) sites are covered by eRRBS. Thus, when compared to the study by Shi *et al*. (39), only 16 genes (*ABCA3*, *ABCG1*, *ANPEP*, *LMNB2*, *GALNT2*, *HMGA1*, *HPCAL1*, *KIF18B*, *PTK2B*, *MID1*, *CENPE*, *SEMA6B*, *TNS3*, *TPX2*, *EPHB4*, *TNFRSF10B*) were noted to be differentially methylated and expressed by both their study and ours. This might be due to variations in experimental protocol, source of PM_2.5_, and methods for DNA methylation analysis (Illumina array vs. eRRBS).

Although PM_2.5_ induced an overall greater number of CpG sites that were hypomethylated compared to hypermethylated, there was increased hypermethylation found among CpG islands and CpG shores. This differential effect suggests that DNA methylation changes induced by PM_2.5_ occur in a locus-specific manner rather than stochastically throughout the genome. Within genes, a greater proportion of hypermethylation was found among promoters and exons whereas hypomethylation was found more among intergenic regions and 3′- and 5′-untranslated regions. This overall greater degree of hypomethylation with certain regions biased toward hypermethylation has been observed in other *in vitro* studies as well (38, 39). Together, these findings further emphasize the non-random, directed nature by which methylation changes occur throughout the genome.

Measurements of DNMT and TET expression after PM_2.5_ exposure revealed that expression of TET1, TET2, and TET3 were elevated after treatment with PM_2.5_ when given repeatedly for seven days. This is consistent with the observation that PM_2.5_ induces an overall greater degree of hypomethylation across the genome, since TET enzymes are recognized to participate in demethylation by the addition of hydroxyl groups to methylcytosine followed by base-excision and repair. The effects of PM_2.5_ on TET expression and DNA hypomethylation are consistent with certain other studies that demonstrate an overall increase in hypomethylation associated with air pollution (41, 42), though the data *in vivo* tend to be mixed. In one, well-designed double cross-over interventional study performed in humans, PM_2.5_ exposure resulted in a decrease in the predominant DNMT and TET isoforms, and this was associated with a decrease in both methylated and hydroxymethylated cytosine (43). Although our data with chronic repeated exposures demonstrated an increase in TET expression, a short-term single exposure was associated with a decrease in TET expression, which would thus be consistent with this recently published finding in humans. The differing effects of PM_2.5_ on TET expression in our study once again highlight the importance of considering duration of exposure in different models. Although several studies demonstrate that PM_2.5_ induces a global level of DNA hypomethylation (41, 42), our study, in addition to these others, identified specific CpG sites that were hypermethylated as well. No changes in DNMT expression were noted after PM_2.5_ exposure in our experiments, and future studies are needed to determine the exact mechanism by which specific loci are targeted for either hyper- or hypomethylation.

Several of the observed changes in DNA methylation correlated with changes in gene expression, though there were also many changes in DNA methylation that were annotated to genes whose expression was not significantly altered. The effects of changes in DNA methylation often depend on the number of CpG loci affected, the magnitude of change, and the location of these changes relative to any given gene, and our study may have identified many methylation changes that were insufficient to effect gene expression by itself. However, these DNA methylation changes may render genes poised for further modulation, and additional studies can test whether these methylation changes affect the sensitivity of cells to subsequent treatment with various mediators, cytokines, or other signals that activate transcription factors. The fact that DNA methylation patterns persist and can be passed during cell division indicates that these PM_2.5_-induced changes may have important implications in long-term health and disease, even after the cessation of PM_2.5_ exposure. Additional studies would be needed to determine the durability and long-term stability of these DNA methylation changes over time.

Our study examined the gene expression and DNA methylation changes induced in BEAS-2B cells, but use of primary airway epithelial cells, from healthy subjects or individuals with airway disease, could produce different findings. We also treated cells directly with PM_2.5_ dissolved in aqueous medium, and experiments performed using an air–liquid interface may produce different results, as suggested by other studies (44, 45). Use of air–liquid interface may also obviate the need to account for how PM_2.5_ is extracted, as varying levels of organic fractions has been shown to independently affect transcriptomic results (13). The lowest concentration we used was 1 µg/cm^2^, which was much lower than many other *in vitro* studies. This may be similar to the low-concentrations of PM_2.5_ that humans are exposed to in developed countries, though calculating the equivalency between *in vitro* and *in vivo* concentrations has its limitations. We utilized PM_2.5_ collected from air monitoring stations in Beijing, China, and similarities between our dataset and others suggest that PM_2.5_, even from different sources, exert common, universal effects. By contrast, transcriptomic and methylomic changes that were uniquely identified in our study could also indicate changes specific to PM_2.5_ from Beijing. DNA methylation changes have been shown to occur in controlled, *in vivo* studies, performed in both rats (39) and humans (27). Although there are limitations to the applicability of our study to real-world, *in vivo* exposures, *in vitro* studies provide the advantage of systematically examining the effects of different concentration and exposure protocols of PM_2.5_ on a single cell type in isolation. Repeated exposures also better approximate real-world conditions in which humans are often exposed to PM_2.5_ on a daily basis. The fact that we observed very distinct gene expression and DNA methylation signatures from cells treated on a repeat vs. single basis highlights the need for more studies utilizing chronic models of exposure. Future studies that examine the upstream signals that are triggered by PM_2.5_, such as generation of reactive oxygen species or activation of nuclear factor kappa B or aryl hydrocarbon receptor, can help shed light on the mechanism(s) by which PM_2.5_ causes these transcriptomic and DNA methylomic changes.

In conclusion, we identified transcriptomic and DNA methylomic changes that occur when bronchial epithelial cells are exposed to PM_2.5_ from Beijing, China. Using RNA-Seq and eRRBS and comparing high vs. low-concentration of PM_2.5_ at both single and repeated exposures, we identified different genome-wide changes at the gene expression and epigenetic level that are unique and distinct from existing literature. These datasets demonstrate the extensive nature by which PM_2.5_ influences the gene transcriptome and epigenome of airway epithelial cells, which may have important implications in long-term health and disease.

## Supplementary data

Supplementary data are available at *EnvEpig* online.

## Supplementary Material

dvaa022_Supplementary_Data
