# Supplementary material for: Effect of concentration and duration of particulate matter exposure on the transcriptome and DNA methylome of bronchial epithelial cells
Source: Environ Epigenet. 2021 Feb 28;7(1):dvaa022. doi: 10.1093/eep/dvaa022 (PMC7928203; doi:10.1093/eep/dvaa022)
Supplement: dvaa022_Supplementary_Data [file dvaa022_supplementary_data.zip › Supplemental Figures.docx]

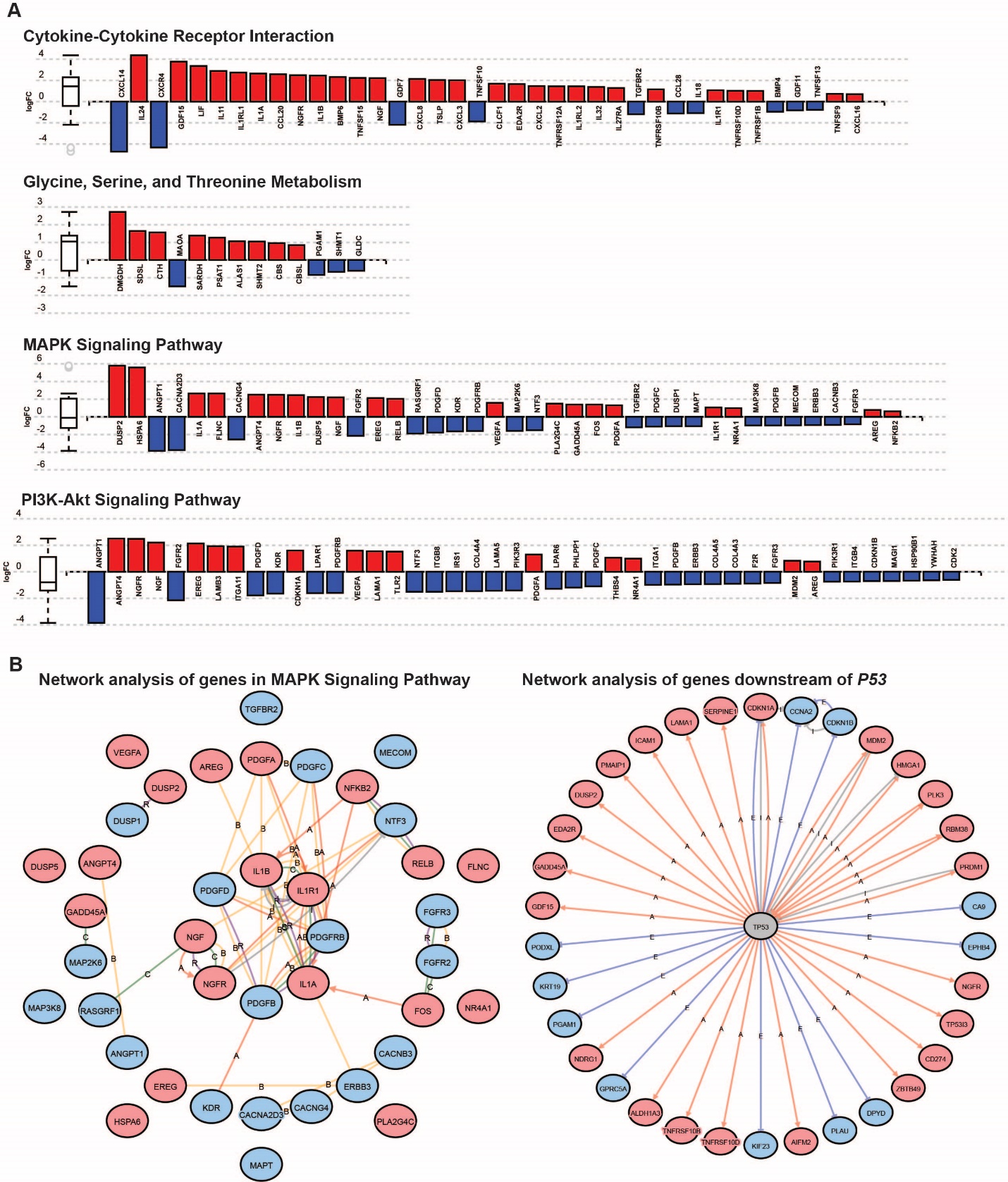


**Supplemental Figure 1 Pathway analysis of differentially expressed genes after 24-h treatment with 30 µg/cm^2^ of PM_2.5_.** A) Pathway analysis identified Cytokine-Cytokine Receptor Interaction, Glycine Serine and Threonine Metabolism, MAPK Signaling Pathway, and PI3K-Akt Signaling Pathway as enriched among differentially expressed genes after high-dose PM_2.5_ treatment. Relative expression of genes in those pathways are shown by log fold change (FC). B) Network analysis of differentially expressed genes in the MAPK Signaling Pathway and genes affected by the upstream mediator, P53.


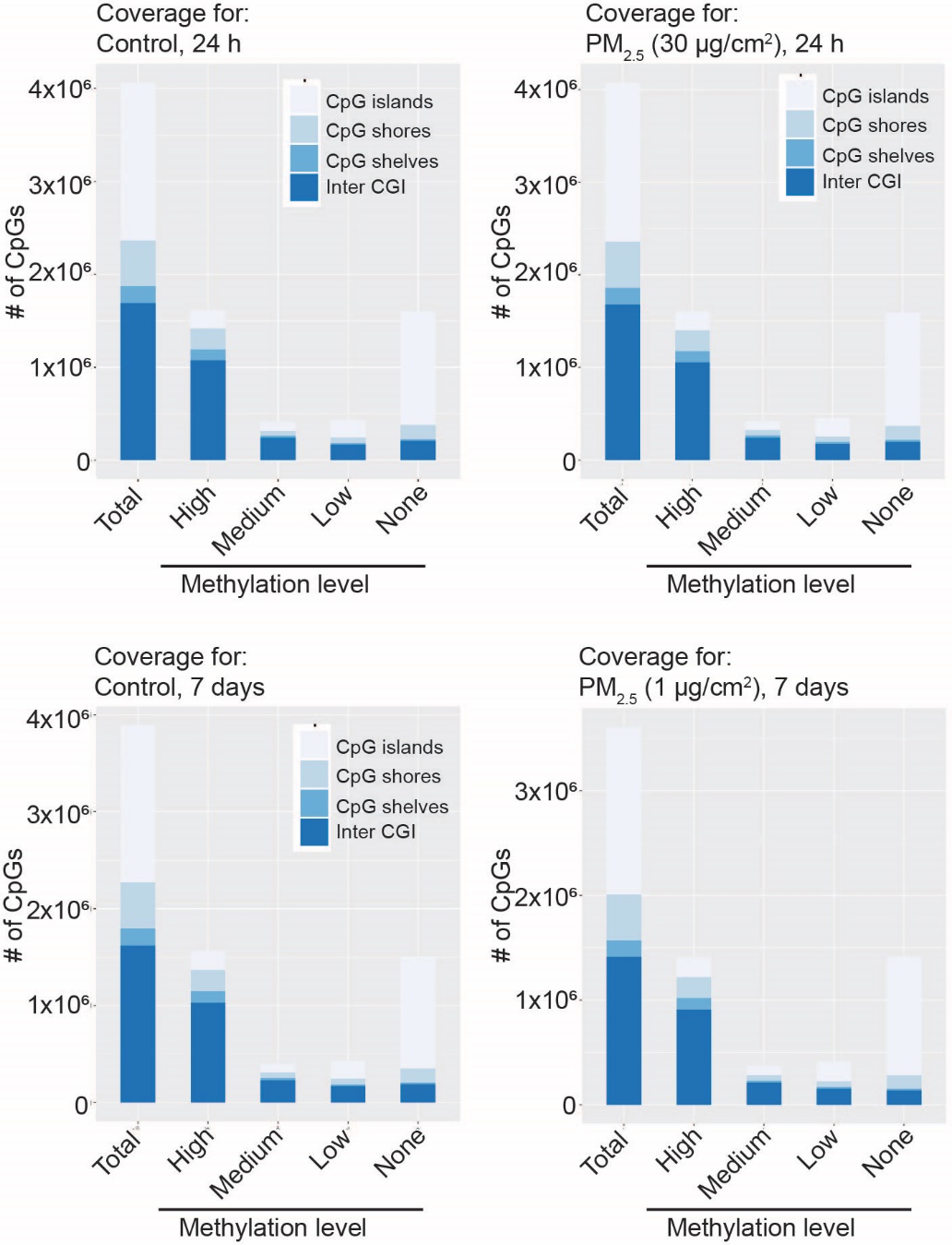


**Supplemental Figure 2 CpG coverage and DNA methylation levels of samples analyzed for enhanced reduced representation bisulfite sequencing (eRRBS).** Samples from different treatment conditions were analyzed for eRRBS. Total number of aligned and annotated CpGs are shown, as well as the distribution of methylation levels (high 66-100% methylation, medium 33-66% methylation, low 5-33% methylation, none < 5% methylation). Included is also the relative distribution of annotations to CpG islands, shores, shelves, or intergenic regions.
